# Scrutinizing the causal link between excited delirium syndrome and restraint: a commentary on ‘The role of restraint in fatal excited delirium: a research synthesis and pooled analysis’ by E.M.F. Strömmer, W. Leith, M.P. Zeegers, and M.D. Freeman

**DOI:** 10.1007/s12024-023-00589-3

**Published:** 2023-02-16

**Authors:** Hans H. de Boer, Judith Fronczek, Melanie S. Archer

**Affiliations:** Victorian Insitute of Forensic Medicine / Dept. of Forensic Medicine of Monash University, 65 Kavanagh Street, Southbank, VIC 3006 Australia

**Keywords:** Excited delirium, Agitated delirium, Restraint, Review, Forensic pathology, Forensic medicine

Dear Editor,

Excited delirium syndrome (ExDS) is a term surrounded by controversy, with currently no consensus on its definition, and considerable doubt about its usefulness as a concept in cause of death examinations. Resolving these issues is a matter of urgency, since the term is almost invariably invoked in complex, emotive, and socially impactful cases. In this context, we would like to comment on the paper of Strömmer et al., published in Forensic Science, Medicine and Pathology in 2020 [1]. In this paper, the authors describe a review of published cases of ExDS and its purported synonym agitated delirium syndrome (AgDS), to analyze the differences in demographics, use of force, drug intoxication, mental health, and survival outcome.

In summary, their analysis of the literature containing case reports and case series identified 120 cases of ExDS and 48 cases of AgDS, in which ExDs was more often fatal (OR of 9.9) and far more often associated with ‘forceful’ or ‘aggressive’ methods of restraint than AgDS (p-values < 0.0001). Their analysis of grouped data of 666 cases of ExDs and 153 cases of AgDs found that 79% of ExDS cases were fatal, but only 3% of individuals with AgDS died.

According to the authors, their findings show that ‘the most probable mechanism driving the association between ExDS and death is the high frequency of aggressive restraint types observed in the ExDS cases’ (p. 683); that ‘there is no evidence to support ExDs as a cause of death in the absence of restraint’ (p. 680); and that ‘when death occurs in an aggressively restrained individual who fits the profile of ExDS or AgDS, restraint-related asphyxia must be considered a likely cause of the death’ (p. 680).

The paper has been cited multiple times in peer-reviewed journals covering forensic pathology [2–4], emergency care [5], psychiatry [6], and general medicine [7]. It is furthermore cited in commentaries [8, 9] and in an influential report of Physicians for Human Rights [10]. In general, these publications interpret Strömmer et al. as if it demonstrates a causal link between restraint and death. This is not unreasonable, given the confidence with which Strömmer et al. interpret their findings and present their conclusions. However, we believe the paper contains major flaws, which severely undermine the conclusions of the authors.

Of the terms excited delirium/ExDS and agitated delirium/AgDS, the former is far more commonly used in the literature of forensic pathology, toxicology, and clinical medicine. But the term AgDS is much rarer and, if seen, is almost invariably used in clinical medicine literature. This is important, since this implies that the literature on ExDS would regularly include fatal cases with detailed discussions of injury, restraint, and mechanism of death. The literature on AgDS however would be more focused on medical treatment and its outcomes in live patients, with far less attention to mortality and restraint. Cases in the AgDS literature would also be more likely to have medically trained personnel as first responders, who are more likely to have sedatives at their disposal, to have the means to take vital observations, and to provide medical treatment where required.

To demonstrate this publication bias, we compiled a table with all references used by Strömmer et al. We then determined whether these publications used the term ExDS or AgDS; whether the described cases were fatal or non-fatal; and what the context of the paper was. For the latter, a choice was made between clinical medicine (focusing on medical management/treatment) and non-clinical (focusing on toxicology or medico-legal issues). The table is provided as Online Resource 1 (See Supplementary Information).

Our data confirms that, despite the suggestion by Strömmer et al., the terms ExDS and AgDS are not used interchangeably. Of the 80 papers used for data extraction, 56 used ExDS and only 9 used AgDs (these numbers differ from those in Strömmer et al., this is further discussed below). All but one of the AgDs papers discussed a clinical medicine context; only 43% of the ExDS papers did so. Four papers explicitly mentioned both terms and eleven papers did not mention ExDs or AgDs at all. We do not know why these fifteen papers were included in the analysis, since another paper that used ‘agitated/excited delirium’ was excluded by Strömmer et al. (see ‘analysis of group data studies’, p. 683). It nonetheless appears that all cases from these fifteen papers were added to the AgDS group. Of these papers, 73% discussed a clinical medicine context.

Based on our findings, it should not be surprising that a diagnosis of ExDS was more often fatal and more often associated with some form of restraint. Or that the AgDS cases were usually non-fatal and more often associated with unknown restraint or sedation (a medical treatment). When Strömmer et al. use this data to infer that ‘the most probable mechanism driving the association between ExDS and death is the high frequency of aggressive restraint types observed in the ExDS cases’ (p. 683), they erroneously infer causation, where only correlation is demonstrated.

It appears that Strömmer et al. were aware of the above shortcoming, because in the introduction, they discuss the different uses of the terms. In the discussion, they also mention the limitation that fatal cases are more often published and are more detailed in terms of type of restraint. However, they fail to fully recognize how the available information differs between ExDS and AgDS, and how their results are confounded by it.

Our review identified multiple other major shortcomings of the paper.

First, our review demonstrated that many of the cited references are totally unsuited to analyze the role of restraint in fatal excited delirium. For instance, many papers discuss the toxidrome and/or pharmacology of drugs, with very limited discussion of other relevant circumstances of the case. Similarly, the discussion of restraint in many clinical papers was limited to one or two general sentences. Some papers are not case descriptions at all, for instance those with a focus on neuropathology [11], the role of oxidative stress in the brain [12], or blood biomarkers [13]. Only a very small portion of the papers provide a comprehensive and detailed discussion needed for the comparative study that Strömmer et al. attempted.

Second, we found that many references were interpreted incorrectly. For the references that were used to source individual cases, the following errors were noted:

- Atherton et al. [14] is cited as if it contributes two cases, but the paper discusses four drug-related deaths of which only one displayed ‘classic CNS-stimulant induced erratic behavior before being found dead’ (no restraint). No diagnosis of ExDS or AgDS was made.
- Byard [15] is cited as if it contributes one case, whilst this paper is an editorial on ExDS without case information.
- Kodikara et al. [16] is cited as if it contributes two cases, but it presents a death due to pulmonary embolism and a death due to cardiac tamponade, initially thought to be ExDS. This paper should therefore have been excluded.
- McDaniel et al. [17] is cited as if it contributes two cases. The paper discusses the clinical effects of GHB withdrawal and states that three of the five patients had severe symptoms, ‘including delirium’. The terms ExDS or AgDS are not used.
- O’Halloran and Frank [18] is cited as if it contributes 20 cases, but according to the paper ‘excited (agitated) delirium, [..] could be interpreted as present in all but 3 of the 21 cases’. In five individuals excited or agitated delirium was included in the cause of death.
- Penders et al. [19] is cited as if it contributes three cases, but the paper only very briefly mentions 16 cases who displayed ‘some degree of agitation’ following use of methylenedioxypyrovalerone (MDPV). No detailed case information is provided.
- Samuel et al. [20] is cited as if it contributes one case, but the paper discusses four cases, of which at least two should have been included (one fatal and one non-fatal case of ExDS).
- Schiavone et al. [12] is cited as if it contributes one case, but the paper does not contain any case information.
- Stratton et al. [21] is cited as if it contributes 18 cases, but the paper describes 214 cases of ExDS with restraint (196 non-fatal, 18 fatal). The non-fatal cases had the same restraint techniques used as the fatal cases. The 18 fatal cases were described in more detail, making them more suitable for case analysis, but by ignoring all survivors, Strömmer et al. misrepresented the original data set.

For the references that were used to source group data, the following errors were noted:

- Baldwin et al. [22] is cited as if it contributes 73 fatal cases of ExDS. Actually, 71 of these 73 individuals survived, and the risk of sudden and unexpected death during arrest in these 73 individuals was estimated to be ‘upward of 9.2%’. In their highlights, Baldwin et al. clearly state that ‘contrary to previous assumptions, not all cases of ExDS result in fatal outcomes’.
- Cole et al. [23] is cited as if it contributes 49 non-fatal cases of AgDS, but the paper actually uses the term ExDS, not AgDS. The 49 patients were ‘agitated’ in the general sense only, and ‘some […] likely had ExDS’.
- Miner et al. [24] is cited as if it contributes 68 cases of non-fatal AgDS. This study describes 43,838 randomly selected patients screened for potential agitation in Emergency Departments, of which 1146 were found to be agitated, and 260 were delirious. Mortality numbers are not given. The authors do not diagnose ExDS or AgDS, but refer in their introduction and discussion to ExDS, not AgDS.
- Strote and Hutson [25] is cited as if it contributes 3 fatal cases of ExDS, but this paper states that of the 37 cases, ‘28 were specifically given a diagnosis of excited delirium’. Only in three cases was it determined to be the cause of death.
- Strote et al. [26] is cited as if it contributes 43 fatal cases of ExDS, but the paper explicitly mentions that ‘no deaths occurred’.

This level of erroneous citation is unacceptable and greatly reduces the paper’s credibility. How these errors have affected the results of Strömmer et al. cannot be determined entirely, especially for the case reports, but for the group studies, it appears that the mortality of ExDS was substantially overestimated and that of AgDS substantially underestimated. Overall, it is clear that Strömmer et al.’s analysis does not accurately represent the published literature.

Third, several papers only focused on fatal cases (e.g. case series on death due to a specific drug, or death during restraint) and subsequently identified one or more cases with features of ExDS. This case selection bias inherent in the literature was present in six of the references used for the case reports [18, 27–31], and ten papers used for the group data analysis [11, 25, 32–39]. Due to circular reasoning, such papers cannot be used to study the lethality of ExDs/AgDS. Since the vast majority (88%) of these papers discussed ExDS, not AgDS, their inclusion especially inflates the mortality of ExDS. In this context, the comment of Strömmer et al. that other researchers ‘manufactured an artificially low ExDS mortality rate’ (p. 685) seems inappropriate.

All in all, Strömmer et al. make the same mistake they attribute to others, namely of basing conclusions on ‘circular reasoning and confounding rather than [on] evidence-based inference’ (p. 684). In our opinion, their paper is fundamentally flawed, and the conclusions are egregiously misleading.

We would like to emphasize that all delirious individuals should receive prompt and adequate medical care, irrespective of the underlying pathology. Also, in deaths temporally related to some form of restraint, the contribution of restraint to the cause of death needs to be rigorously scrutinized. In many such cases, restraint may be a significant contribution, or even the sole cause of death. However, by suggesting that restraint is the main fatal mechanism in all cases with an ExDS-type presentation and some form of restraint, Strömmer et al. deny the complex, multifactorial mechanisms by which death can occur, and the variety that exists between cases.

### Supplementary Information

Below is the link to the electronic supplementary material.

Supplementary file1 (PDF 649 KB)

## Data Availability

The data supporting the findings of this study are available within the article and its supplementary materials.
