# Supplementary material for: Scrutinizing the causal link between excited delirium syndrome and restraint: a commentary on ‘The role of restraint in fatal excited delirium: a research synthesis and pooled analysis’ by E.M.F. Strömmer, W. Leith, M.P. Zeegers, and M.D. Freeman
Source: Forensic Sci Med Pathol. 2023 Feb 16;19(4):613–6. doi: 10.1007/s12024-023-00589-3 (PMC10752912; doi:10.1007/s12024-023-00589-3)
Supplement: Supplementary file 1 — Supplementary file1 (PDF 649 KB) [file 12024_2023_589_MOESM1_ESM.pdf]

Review of the references used for data extraction by Strömmer *et al.*,

* For full references, see Strömmer *et al.*
* For 'context' a difference was made between 'clinical' (relating to clinical medicine) and 'non-clinical' (relating to toxicology or medico-legal issues)
* 'Strömmer N' and 'Strömmer Fatalities' indicate number of cases and fatalities according to Strömmer *et al.* ; 'Reviewed N ' and 'Reviewed Fatalities' indicate number of cases and fatalities according to our review
* Red squares indicate differences between Strömmer *et al.* and our review.

REFERENCES FOR THE CASE ANALYSIS					Strömmer	Strömmer	Reviewed	Reviewed	
**First author**	**Publishing journal**	**Year**	**Term used**	**Context**	**N**	**Fatalities**	**N**	**Fatalaties**	**Paper summary**
Aberegg	J Lung Pulm Respir Res.	2014	ExDS	Clinical	1	N/a	1	0	Clinical management of a non-fatal case of ExDS of unknown cause
Alciati	J Psychoactive Drugs.	1999	Neither	Clinical	3	N/a	3	0	Clinical case series of three non-fatal cases of delirium following MDMA overdose
Atherton	J Forensic Sci.	2019	ExDS	Non-clinical	2	N/a	1	0	Toxicological case series of 4 deaths related to n-Ethyl Pentyoil
Benzer	N Engl J Med.	2013	AgDS	Clinical	1	N/a	1	0	Clinical case report of a non-fatal "bath salt" intoxication
Blaho	J Clin Forensic Med.	2000	ExDS	Non-clinical	2	N/a	2	1	Toxicological Case series to discuss the half-life of cocaine in hyperthermic patient, using one fatal and one non-fatal case of cocaine-induced ExDS
Bozeman	J Emerg Med.	2013	ExDS	Clinical	1	N/a	1	0	Clinical case report on the diagnosis of long-QT syndrome in a non-fatal case of ExDS
Bunai	Legal Med.	2008	ExDS	Non-clinical	1	N/a	1	1	Case report of fatal hyperthermia in a case of ExDS with restraint
Burnett	Prehospital Emerg Care.	2012	ExDS	Clinical	1	N/a	1	0	Clinical case report of laryngospasm after intramuscular ketamine administration in a non fatal case of ExDs
Byard	J Forensic Sci.	2016	ExDS	Non-clinical	1	N/a	1	1	Forensic case report of head trauma in a fatal drug-induced ExDS, without restraint
Byard	Forensic Sci Med Pathol.	2017	ExDS	Non-clinical	1	N/a	0	0	Forensic pathologist commentary on the issues surrounding ExDS. No case described
Corstens	J Forensic Legal Med.	2018	ExDS	Clinical	1	N/a	1	0	Clinical Case report of non-fatal ExDs after GHB withdrawal
Daugherty	Law Enf. Man. Inst. of Texas	2012	ExDS	Non-clinical	2	N/a	2	2	Thesis from a police commander on acceptance and recognition of ExDS by law enforcement. Includes 1 fatal case of cocaine-induced ExDS
Debelmas	Radiology	2018	ExDS	Clinical	1	N/a	1	0	Clinical radiology case description of non-fatal drug-induced ExDS
Desharnais	Forensic Sci Med Pathol.	2017	ExDS	Non-clinical	1	N/a	1	1	Toxicological case report of fatal MDPV-induced ExDS
Downes	Clin Toxicol.	2015	AgDS	Clinical	1	N/a	1	0	Clinical case report of non-fatal phenibut-induced ExDS
Dyer	Ann Emerg Med.	2001	Neither	Clinical	8	N/a	8	1	Clinical case series of 7 non-fatal and 1 fatal case of GHB withdrawal
Feeney	J Natl Med Assoc.	2010	AgDS	Clinical	1	N/a	1	1	Clinical case report of a non-fatal Taser-induced agitated state
Fishbain	Ann Emerg Med.	1981	Neither	Clinical	1	N/a	1	1	Clinical case report on the medical management (misdiagnosis) of fatal cocaine-induced delirium
Ho	Prehospital Emerg Care.	2012	ExDS	Clinical	2	N/a	2	1	Clinical case series of two non-fatal ExDS, succesfully treated with pre-hospital ketamine
Imam	J Emerg Med.	2013	Neither	Clinical	6	N/a	6	1	Clinicial case series on the clinical aspects and management of 1 fatal case and 5 non-fatal instances of bath salt toxicity (not diagnosed with ExDS or AgDs)
Jovel	J Forensic Sci.	2014	ExDS	Non-clinical	1	N/a	1	0	Toxicological case report of non-fatal tryptamine 5-eO-DALT-induced ExDS
Kasick	Am J Drug Alcohol Abuse.	2012	Neither	Clinical	2	N/a	2	0	Clinical case series of two non-fatal cases of "bath salt" toxicity
Kennedy	J Correct Heal Care.	2017	ExDS	Clinical	1	N/a	1	1	Clinical case report of delayed death after ExDS
Kesha	J Forensic Sci.	2013	ExDS	Non-clinical	1	N/a	1	1	Toxicological case report of fatal MDPV-induced ExDs
Kiely	J Anal Toxicol.	2009	ExDS	Non-clinical	1	N/a	1	1	Toxicological case report of fatal methamphetamine-induced ExDS
Kodikara	Legal Med.	2012	ExDS	Non-clinical	2	N/a	0	0	Forensic pathology case series of two cases which were initially thought to be fatal ExDS, but pulmonary embolism and cardiac tamponade at autopsy
Kowalski	Pediatr Emerg Care.	2017	AgDS	Clinical	5	N/a	5	0	Clinical case series on ketamine treatment in 5 cases of non-fatal AgDS
Kristofic	J Anal Toxicol.	2016	ExDS	Non-clinical	1	N/a	1	1	Toxicological case report of fatal 25C-NBOMe-induced ExDS
Kunz	Forensic Sci Int	2018	ExDS	Non-clinical	1	N/a	1	1	Forensic pathology case report on death during restraint in man with ExDS (deemed manslaughter)
Labay	Forensic Sci Int.	2016	ExDS	Non-clinical	2	N/a	2	2	Retrospective analysis of 25 deaths due to synthetic cannibinoids, including two deemed ExDS
Lucena	Eur Heart J.	2010	ExDS	Clinical	3	N/a	3	3	Prospective analysis of cocaine-induced sudded death, includes 3 fatal ExDS cases.
Lusthof	Forensic Sci Int.	2011	ExDS	Non-clinical	1	N/a	1	1	Toxicological case report of fatal mephedrone-induced ExDS
Maher	Can J Emerg Med.	2014	ExDS	Clinical	1	N/a	1	0	Clinical case report of a succesful (non-fatal) resuscitation in ExDS
Mash	J Chem Neuroanat.	2000	ExDS	Non-clinical	8	N/a	8	8	Neuropathological study on the effects of cocaine in ExDS vs. non-ExDS cases
McDaniel	J Psychoactive Drugs	2001	Neither	Clinical	2	N/a	3	0	Clinical case series discussing the clinical features of five non-fatal cases of GHB withdrawal
Menaker	J Emerg Med.	2011	AgDs	Clinical	1	N/a	1	0	Clinical case report of non-fatal cocaine-induced AgDS with hyperthermia
Miller	Restraint Asphyxia Newz Dir.	1998	ExDS	Clinical	1	N/a	1	0	Clinical case report of a non-fatal case of ExDS with restraint.
Mirchandani	Am J Forensic Med Pathol.	1994	AgDS	Non-clinical	4	N/a	4	4	Forensic pathology case series of 4 cases of sudden death after cocaine-induced AgDS with restraint
Morrison	Med Sci Law.	2001	ExDS	Non-clinical	1	N/a	1	1	Forensic pathology case report of a fatal psychotic ExDS with restraint
Murray	J Med Toxicol.	2012	ExDS	Non-clinical	1	N/a	1	1	Toxicological case report of fatal MDPV-induced ExDs.
O'Halloran	Am J Forensic Med Pathol.	1993	ExDS	Non-clinical	11	N/a	11	11	Forensic pathology case series of 11 fatal ExDS cases with restraint
O'Halloran	Am J Forensic Med Pathol.	2000	Both	Non-clinical	20	N/a	18	18	Forensic pathology case series on death during prone restraint, 18 cases deemed to have ExDs or AgDs, 5 times included in cause of death
Park	Prehospital Emerg Care.	2001	AgDS	Clinical	2	N/a	2	2	Clinical case series describing two fatal AgDS cases on an emergency department
Penders	Am J Drug Alcohol Abuse.	2012	ExDS	Non-clinical	3	N/a	0	0	Toxicological discussion of MPDV-induced delirium
Pestaner	Am J Forensic Med Pathol.	2003	Neither	Non-clinical	2	N/a	2	2	Toxicological case series on deaths during the arrest of two cases of phyncyclidine-induced delirium
Plush	J Intensive Care Med.	2015	AgDS	Clinical	1	N/a	1	0	Clinical case report describing the clinical management of a non-fatal cocaine-induced delirium
Pritchard	Air Med J.	2014	Neither	Clinical	1	N/a	1	0	Clinical case report on the use of ketamine to treat a non-fatal case of drug-induced psychosis
Rayamane	J Indian Soc of Toxicology	2015	ExDS	Non-clinical	2	N/a	2	2	Toxicological case series of two cases of acute death during alcohol withdrawal, deemed to be ExDS
Reichmuth	BMJ Case Rep.	2015	Neither	Clinical	1	N/a	1	1	Clinical case report of non-fatal delirium due to baclofen overdose
Ruttenber	Am J Forensic Med Pathol.	1999	ExDS	Clinical	1	N/a	1	1	Retrospective case-control study between fatal ExDS and fatal cocaine-induced rhabdomyolysis, introduced by 1 fatal case, not classified as ExDS or AgDS
Samuel	Neuropsychiatr Dis Treat.	2009	Both (ExDS preferred)	Clinical	1	N/a	2	1	Clincial case series and review on ExDS
Scaggs	Prehosp Disaster Med.	2016	ExDS	Clinical	7	N/a	7	0	Retrospective clinical case series of 7 effective ketamine treatments in pre-hospital setting for (non-fatal) ExDS
Schiavone	Toxicol Lett.	2016	ExDS	Non-clinical	1	N/a	0	0	Literature review on contribution of brain NADPH oxidase derived oxidative stress in the development of cocaine-induced ExDS
Shields	J Forensic Sci.	2015	ExDS	Non-clinical	1	N/a	1	1	Forensic pathology case report of sudden death due to cocaine-induced ExDS
Śliwicka	J Forensic Leg Med.	2019	ExDS	Non-clinical	3	N/a	3	3	Forensic pathology case series of three fatal cases of ExDS
Storey	St Louis Univ Law J.	2012	ExDS	Non-clinical	1	N/a	1	1	Legal thesis on ExDS, introduced by a case report of fatal cocaine-induced ExDS
Stratton	Ann Emerg Med.	1995	ExDS	Clinical	2	N/a	2	2	Clinical case series of two deaths during restraint for drug-induced ExDS
Stratton	Am J Emerg Med.	2001	ExDs	Clinical	18	N/a	216	18	Clinical case-control study of differences between fatal vs. non-fatal cases of restraint for ExDS
Sztajnkrycer	Emerg Med Serv.	2005	Both (ExDS preferred)	Clinical	1	N/a	1	1	Clinical literature review of cocaine-induced ExDS, introduced by case report of 1 fatal case
Wetli	J Forensic Sci.	1985	ExDs	Non-clinical	7	N/a	7	7	Forensic pathology case series 7 cases of fatal cocaine-induced ExDS
Wiebe	Paediatr Child Heal.	2008	Neither	Non-clinical	4	N/a	4	4	Toxicologcal case series of 4 children with non-fatal "Angel's trumpet" poisoning. Does not use term Excited Delirium

REFERENCES FOR THE GROUP ANALYSIS					Strömmer	Strömmer	Reviewed	Reviewed	
**First author**	**Publishing journal**	**Year**	**Term used**	**Context**	**N**	**Fatalities**	**N**	**Fatalaties**	**Paper summary**
Baldwin	J Forensic Legal Med.	2016	ExDS	Non-clinical	73	73	73	2	Analysis of 5962 instances of use of force by police to identify features of ExDS. 71 non-fatal and 2 fatal cases with more than 6 features of ExDs
Cole	Am J Emerg Med.	2018	ExDS	Clinical	49	0	?	0	Clinical study of the effectiveness of ketamine as treatment for prehospital agitation. 49 cases with 'profound agitation'. Only one fatal case with an unrelated cause of death
Ezaki	Am J Drug Alcohol Abuse.	2016	ExDS	Non-clinical	2	2	2	2	Toxicological case series on 61 cases of fatal synthetic cannabinoid overdose, with two as ExDS as cause of death
Grant	Am J Forensic Med Pathol.	2009	ExDs	Non-clinical	21	21	21	21	Review of 62 deaths in police custody with features of ExDS, but only 21 with fatal ExDS
Gray	Med J Aust.	2007	AgDS	Clinical	31	0	31	0	Study on clinical characteristics of amphetamine-related presentations to emergency departments: 156 non-fatal cases of which 31 cases with 'sympathomimetic agitated delirium'
Hall	J Forensic Legal Med.	2015	ExDS	Non-clinical	86	1	86	1	Study on the prevalence of ExDS in 4828 'use of force' police encounters of which 86 individuals (1 fatal) had 6 or more features of ExDS
Ho	West J Emerg Med.	2009	ExDS	Non-clinical	102	102	102	102	Study on arrest-related deaths. 162 cases, of which 102 displayed 'erratic behaviour', based on media sources (no medical diagnoses)
Li	J Emerg Med.	2019	ExDS	Clinical	31	0	31	0	Clinical study to evaluate ketamine as a treatment for ExDS. 31 non-fatal cases
Mash	Forensic Sci Int.	2009	ExDs	Non-clinical	90	90	90	90	Retrospectve analysis of biomarkers in autopsy brains of 90 fatal ExDS cases, compared to 30 control brains
Michaud	J Forensic Legal Med.	2016	ExDS	Non-clinical	35	35	35	35	Retrospective analysis of restraint related deaths with (n=35) and without (n=6) ExDS
Miner	Ann Emerg Med.	2018	Neither	Clinical	68	0	260	?	Prospective study on characteristics and prevalence of agitated patients in emergency departments: 43 838 patient included, of which 1 146 agitated, and 260 met criteria for delirium
Mo	West J Emerg Med.	2020	ExDs	Clinical	37	0	37	0	Retrospective analysis of ketamine treatment for 'severe agitation/excited delirium' in emergency departments
Pollanen	Can Med Ass J	1998	ExDs	Non-clinical	21	21	21	21	Analysis of 21 cases of fatal ExDS
Ross	Modern Pathol.	1998	ExDS	Non-clinical	61	61	61	61	Analysis of 61 cases of fatal ExDS in police custody
Ruttenber	J Forensic Sci.	1997	ExDS	Non-clinical	58	58	58	58	Toxicological case-control study of 58 fatal cocaine-induced ExDS vs 125 fatal cocaine overdose without ExDS
Southall	J Forensic Legal Med.	2008	Both	Non-clinical	24	24	24	24	Analysis of 45 cases of death in police custody, with 19 fatal ExDS and 5 fatal AgDS
Strote	Prehospital Emerg Care.	2006	ExDs	Non-clinical	3	3	28	3	Analysis of 37 Taser-related deaths, 28 with ExDs, of which 3 as cause of death
Strote	Am J Emerg Med.	2014	ExDS	Clinical	43	43	43	0	Clinical analysis of prevalence of medical diagnoses and restraint in 43 individuals with non-fatal ExDS
Vilke	J Forensic Legal Med.	2019	ExDs	Clinical	21	0	21	0	Prospective study on biomarkers in ExDS, comparing 30 controls, 25 agitated and 21 ExDS patients
